# Comparative Assessment of Pivotal Trials Supporting the Indication Approvals of Innovative and Modified New Anticancer Drugs in China, 2016–2022

**DOI:** 10.34133/hds.0263

**Published:** 2025-05-02

**Authors:** Lixia Fu, Ruifen Xue, Jie Chen, Guoshu Jia, Xiaocong Pang, Yimin Cui

**Affiliations:** ^1^ Institute of Clinical Pharmacology, Peking University First Hospital, Beijing, China.; ^2^ Beijing Key Laboratory of Clinical Pharmacology and Translation of Innovative Drugs, Beijing, China.; ^3^ Department of Pharmacy Administration and Clinical Pharmacy, School of Pharmaceutical Sciences, Peking University, Beijing, China.; ^4^ Department of Pharmacy, Peking University First Hospital, Beijing, China.; ^5^ State Key Laboratory of Natural and Biomimetic Drugs, School of Pharmaceutical Sciences, Peking University, Beijing, China.

## Abstract

**Background:** Since the launch of drug regulatory reform in 2015, China has substantially increased the availability of new cancer therapies. However, the efficacy evidence criteria for modified new anticancer drugs have not been evaluated. This cross-sectional study aimed to assess the pivotal trials supporting the indication approvals of innovative and modified new chemical anticancer drugs in China. **Methods:** The characteristics of indications, regulatory aspects, and pivotal trial designs were extracted and described. The primary efficacy endpoints of the pivotal clinical trials, including overall survival (OS) and progression-free survival (PFS), were quantitatively assessed by meta-analysis. **Results:** Between 2016 and 2022, 77 cancer therapeutics for 107 indications were approved in China based on 128 pivotal trials. Among the 107 indications, 64 (59.8%) were classified as innovative anticancer drugs, and 43 (40.2%) as modified new anticancer drugs. The study found that pivotal trials for innovative approvals tended to be single-arm trials, while modified approvals were more likely to employ randomized clinical trials with larger sample sizes and rigorous designs. Despite innovative drugs often receiving more expedited regulatory designations, there were no statistically significant differences in clinical benefit of OS or PFS outcomes between innovative and modified approvals. **Conclusions:** These results suggest that the current regulatory framework may prioritize the speed of approval for innovative drugs over the strength of supporting evidence. These findings align with the strategic trends of pharmaceutical companies and regulatory inclinations that aim to expedite the approval of innovative anticancer drugs with a high unmet need, thereby accelerating patients’ accessibility to treatment.

## Introduction

Long regulatory review time and a large backlog of new drug applications (NDAs) at the China Food and Drug Administration (CFDA, now referred to NMPA, National Medical Products Administration) delayed the accessibility to new drugs for patients in China [1]. As the conflict between the public’s urgent healthcare needs and the sluggish progress of drug review and approval continued to intensify, the delay in drug launch has long troubled health policymakers, which triggered the regulatory reform in 2015 [2,3]. In August 2015, the State Council of the People’s Republic of China released the landmark document “Opinions on the Reform of Review and Approval System for Drugs and Medical Devices” (State Council [2015] No.44), initiating a comprehensive reform plan [4]. The No.44 Opinion introduced the categorization of drugs into new and generic drugs, further classifying new drugs into innovative and modified new drugs. Thereafter, a series of regulatory reform policies and regulations were issued by the Chinese government. One of the most important reform measures involved altering the drug registration classification through issuing the documents of “Work Plan for the Reform of Chemical Drug Registration Classification” (NMPA [2016] No.51) [5] in March 2016, which redefined the classification of chemical drug registration. This classification was further defined in the “Provisions for Drug Registration” [6] in March 2020 and the “Requirements for Registration Classification and Application Dossiers of Chemical Drug*s”* [7] in June 2020 (Table S1).

With the expedited progress of drug review and approval, the quantities of new drugs have dramatically increased in China, especially between 2017 and 2021 [8]. The implementation of various policies aimed at encouraging innovation has extensively promoted the development of innovative drugs [9]. The majority of these new drugs were intended for the treatment of cancer, and the quantities of new anticancer drug approvals have increased substantially over the past 5 years in China [10,11]. Meanwhile, the number of multi-indications, new reformulations, or new dosage forms of anticancer drugs approved in the market is also increasing driven by the regulatory reform [12,13]. The clinical benefit varies among different indications [14] or different dosage forms of the same drug. Drugs that have their structure, dosage form, formulation and process, route of administration, and indications optimized on the basis of known active ingredients are classified as modified new drugs in China [7]. The detailed concepts corresponding to the chemical drug registration classification category are outlined in Table S2.

The increasing healthcare cost has been a great challenge to China’s health care system, which is highlighted by the frequent negative press coverage [15]. A study conducted by Zhang et al. [16] showed that the catastrophic health expenditure (CHE) rate in China has been rising over the past 20 years, with a concurrent upward trend in out-of-pocket (OOP) health expenditures. In particular, the cost of anticancer drug treatments has more than doubled and experienced an annual increase over the past 5 years [17,18]. The association between costs and clinical value of anticancer drugs has become a critical issue around the world [14,18–25].

The clinical value and the quality of evidence often vary among different indications. In China, multi-indication drugs adhere to a single drug pricing policy [26], which is similar to the approach in the United States [14]. It is critical to understand the regulatory characteristics and their potential impact on the quality of evidence supporting the approvals for innovative and modified new anticancer drugs. This is particularly important for patients, healthcare providers, and policymakers to evaluate the value of new anticancer drugs. There are numerous studies on the topic of initial and supplementary indication approval of new anticancer drugs globally [19,27–29]. However, previous studies mainly focused on the clinical evidence supporting the approval of novel anticancer drugs in China [30,31]. Limited information exists regarding the pivotal clinical trial evidence supporting the approval of modified new anticancer drugs.

To our knowledge, no study has characterized the pivotal clinical trial evidence supporting both innovative and modified anticancer approvals by the NMPA. Whether regulatory characteristics and the clinical trial evidence base required for modified cancer therapies differ from those for innovative approvals remain unknown. In this study, we evaluated all new anticancer drugs approved by the NMPA through the new registration classification from 2016 to 2022, to determine whether the pivotal clinical trial features differed among innovative and modified new anticancer drugs.

## Methods

### Data source and selection criteria

**Fig. 1.**
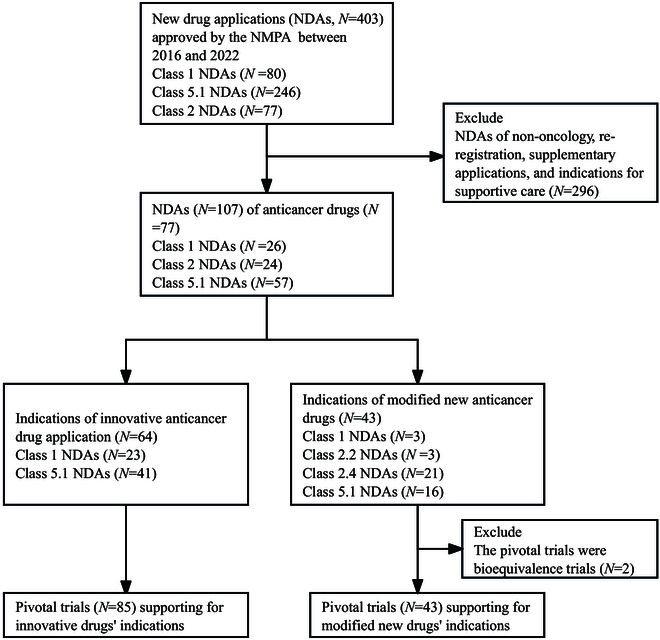
Flowchart of NMPA-approved new anticancer drugs, indications, and pivotal trials between 2016 and 2022 identified in this study.

We obtained all NDAs approved from 2016 January 1 to 2022 December 31 via the new registration classification from the commercial database Pharmacodia [32] and cross-checked the data through the Center for Drug Evaluation (CDE, NMPA) official website (Fig. 1). To maintain precision, different strengths within a single approved product were treated as one approved NDA. The initiation of our database in 2016 aligns with the promulgation date of NMPA [2016] No.51 [5], marking the implementation of the new drug registration classification. In addition, the regulatory review documents and labels of new drugs have been made publicly available by the CDE since July 2016 [33]. According to the World Health Organization’s Anatomic Therapeutic Classification (ATC) system [34] and the NDAs’ indication descriptions in the labels, the anticancer drug list was identified, which comprised both innovative and modified new anticancer drugs, excluding NDAs related to drug re-registration, supplementary applications, and indications for supportive care (Fig. 1).

### Data extraction

Regulatory review documents and drug labels were retrieved from publicly available database provided by CDE [35] to extract key characteristics of pivotal trials for each drug, including indications, clinical and regulatory specifics, and trial features for all eligible clinical studies. Additionally, we supplemented our data collection with information from the website of clinicaltrials.gov or chinadrugtrials.org.cn when the corresponding information was not disclosed in the review documents. Two investigators independently validated the pivotal trials, and any disagreements were resolved by consensus. In cases of disparities in pivotal trial data between regulatory documents and other sources, we prefer to extract data from regulatory documents, as this information had been identified and reviewed by regulators.

### Indication and regulatory characteristics

Based on information from regulatory review documents of NDA approvals, we classified each NDA by the Class of registration (see Table S2) and delineated the approved indications for each drug, categorizing them into solid tumors or hematological malignancies. Further categorization was conducted based on drug mechanisms, including chemotherapy, radiotherapy drugs, hormone therapy, targeted therapy, and immunotherapy. All sample anticancer drug indications were categorized into first-line, second-line, third-line and later-line, adjuvant, and maintenance therapies.

Since 2007, China has developed several expedited programs [11], including special review and approval, priority review and approval, and conditional approval, which are used to shorten the review time and expedite the approval for therapeutics intended to address unmet clinical needs for serious or life-threatening conditions [6] (Table S3). To understand the distribution patterns of innovative drugs and modified new drugs receiving these special regulatory designations, we extracted this information from regulatory review documents. The availability of expedited regulatory programs varied across years; the special review program was only available from 2007 to 2020, while priority review and conditional approval were introduced separately in 2015 and 2017 by the NMPA. Breakthrough therapy designation was not identified in our study because it is applicable during the Investigational New Drug Application (IND) period.

### Characteristics of pivotal trials

For each drug, the review documents explicitly indicated the clinical trials designated as pivotal. We extracted data on trial design, including randomization, blinding, and number of treated patients (overall and intervention group); trial control, such as active treatment, placebo control, or no control; primary endpoints, including overall survival (OS), progression-free survival (PFS), and objective response rate (ORR); and trial results, such as hazard ratio (HR). To determine the time of review and the duration of each trial, we reviewed the regulatory document and searched domestic and international clinical trial website. The submission and approval dates were used to calculate the review time. The actual start date and primary completion date of each trial, as reported on ClinicalTrials.gov and chinadrugtrials.org.cn, were used to calculate the trial duration. Studies that only reported pharmacokinetic comparisons, such as bioequivalence studies, were excluded from the analysis. Pivotal clinical trials supporting approvals for different indications were counted only once, and any repeated trials were excluded.

### Statistical analysis

Descriptive statistics was used to characterize the sample of new anticancer drugs and features of their approved indications and regulation, and examined differences in indication and pivotal trial characteristics. Medians [interquartile ranges (IQRs)] were used for continuous variables, and counts and percentages for categorical variables. We used the χ^2^ test for the categorical variables, and the Mann–Whitney *U* test for the continuous variables. To prevent underestimating cases with missing data, the aggregated analyses by indication included only those with complete reporting of pivotal clinical trial characteristics. Analyses were stratified by origin, cancer type, and the use of expedited programs. To evaluate the association between new anticancer drug types and treatment outcomes (OS or PFS) and evaluate the efficacy of innovative and modified new drugs, we used the method of meta-regression. We did not conduct a meta-analysis of ORR because of the very small proportion of RCTs (randomized clinical trials) using ORR. Heterogeneity was reported on the basis of the *I*^2^ statistic. This result may provide evidence-based insights that can guide clinical decisions and policy-making regarding the use or pricing of these drugs.

Statistical analyses were conducted using Microsoft Excel version 2021 software (Microsoft Corp.), IBM SPSS version 27 0 (IBM Corp.), R version 4.3.2 (R Project for Statistical Computing), and RStudio software version 2023.09.1 (RStudio, PBC). A 2-tailed *P* value of <0.05 was considered statistically significant.

## Results

### Indication and regulatory characteristics

Between 2016 and 2022, the NMPA approved 77 new anticancer drugs for 107 indications (Fig. 1). Of these 107 indications, 64 (59.8%) were categorized as innovative anticancer drugs, and 43 (40.2%) as modified new anticancer drugs. The number of approvals increased from 0 in 2016 to 30 in 2021. Among the 107 indications, 41 (38%) were domestic and 66 (62%) were imported. The majority (80/107, 75%) of indications were for the treatment of solid cancer. The most common cancer type was lung cancer (28%), followed by lymphoma (10%) and breast cancer (9.3%). Innovative drugs were significantly more likely to receive priority review (84.4% versus 60.5%, *P* = 0.005) and had a higher proportion to receive special review (20.3% versus 11.6%, *P* = 0.24) and conditional approval (48.4% versus 30.2%, *P* = 0.06) compared to modified new drugs. With regard to drug mechanism class, targeted therapies were the main drug class for both innovative and modified new cancer indications. There was no significant difference in the distribution of innovative and modified new indications across cancer lines, with both predominantly in the first and second lines. The review time of modified new indications was shorter than for innovative indications (median [IQR], 9.9 [8.4 to 11.9] months versus 12.6 [9.6 to 15.9] months, *P* = 0.009) (Table 1).

**Table 1.** Characteristics of innovative and modified indications approved by the NMPA from 2016 to 2022. Note: Sums may not total to 100% because of rounding.

Characteristic	Indications, no. (%)			*P* value
	All (*n* = 107)	Innovative drugs (*n* = 64)	Modified new drugs (*n* = 43)	
Approval year				0.41
2016	0 (-)	0 (-)	0 (-)	
2017	4 (3.7)	3 (4.7)	1 (2.3)	
2018	12 (11.2)	10 (15.6)	2 (4.7)	
2019	10 (9.3)	7 (10.9)	3 (7.0)	
2020	22 (20.6)	12 (18.8)	10 (23.3)	
2021	30 (28.0)	15 (23.4)	15 (34.9)	
2022	29 (27.1)	17 (26.6)	12 (27.9)	
Origin				0.15
Domestic	41 (38.3)	21 (32.8)	20 (46.5)	
Imported	66 (61.7)	43 (67.2)	23 (53.5)	
Cancer type				0.20
Solid	80 (74.8)	45 (70.3)	35 (81.4)	
Hematologic	27 (25.2)	19 (29.7)	8 (18.6)	
Special regulatory program^a^				
Priority review	80 (74.8)	54 (84.4)	26 (60.5)	0.005
Special review	18 (16.8)	13 (20.3)	5 (11.6)	0.24
Conditional approval	44 (41.1)	31 (48.4)	13 (30.2)	0.06
Drug mechanism class				0.30
Chemotherapy	15 (14.0)	7 (10.9)	8 (18.6)	
Hormone therapy	4 (3.7)	3 (4.7)	1 (2.3)	
Immunotherapy	2 (1.9)	1 (1.6)	1 (2.3)	
Others	2 (1.9)	0 (-)	2 (4.7)	
Radiotherapy	2 (1.9)	2 (3.1)	0 (-)	
Targeted therapy	82 (77)	51 (79.7)	31 (72.1)	
Review time (months), median (IQR)	11.2 (8.7–14.3)	12.6 (9.6–15.5)	9.9 (8.4–11.8)	0.009
<6		2 (3.1)	2 (4.7)	
6–12		28 (43.8)	31 (72.1)	
12–18		20 (31.3)	5 (11.6)	
>18		14 (21.9)	5 (11.6)	
Lines of therapy				0.05
1st	45 (42.1)	30 (46.9)	15 (34.9)	
2nd	36 (33.6)	19 (29.7)	17 (39.5)	
3rd or later	9 (8.4)	8 (12.5)	1 (2.3)	
Adjuvant	9 (8.4)	2 (3.1)	7 (16.3)	
Maintenance	2 (1.9)	1 (1.6)	1 (2.3)	
Others	6 (5.6)	4 (6.3)	2 (4.7)	
Most common cancer type				0.60
Lung cancer	30 (28.0)	16 (25.0)	14 (32.6)	
Lymphoma	11 (10.3)	8 (12.5)	3 (7.0)	
Breast cancer	10 (9.3)	7 (10.9)	3 (7.0)	
Prostate	7 (6.5)	5 (7.8)	2 (4.7)	
Ovary	7 (6.5)	4 (6.3)	3 (7.0)	
Thyroid	7 (6.5)	2 (3.1)	5 (11.6)	
Multiple myeloma	5 (4.7)	3 (4.7)	2 (4.7)	
Colorectal	4 (3.7)	3 (4.7)	1 (2.3)	
Gastrointestinal	3 (2.8)	2 (3.1)	1 (2.3)	
Leukemia	3 (2.8)	1 (1.6)	2 (4.7)	
Acute myeloid leukemia	3 (2.8)	3 (4.7)	0 (-)	
Others	17 (15.9)	10 (15.6)	7 (16.3)	

^a^ Drugs may qualify for more than one special regulatory program.

### Characteristics of pivotal trials

The study identified 128 pivotal trials for the 107 cancer indications, all of which were explicated in the regulatory review documents or labels. The 64 innovative indication approvals were supported by 85 pivotal trials, while the 43 modified indication approvals were supported by 43 pivotal trials, respectively. Approximately 48.2% of innovative indication approvals were supported by at least 2 pivotal trials, while this proportion in modified approvals was only 11.6%. The number of pivotal trials per indication was significantly different between innovative and modified indications (1 pivotal trial, 51.8% versus 88.4%; 2 pivotal trials, 38.8% versus 11.6%; 3 pivotal trials, 9.4% versus 0%; *P* = 0.001). Compared with innovative trials, modified trials were more likely to be RCTs rather than single-arm trials (88.4% versus 52.9%, *P* < 0.001). Additionally, they were more frequently randomized (86.0% versus 54.1%, *P* < 0.001) and double-blinded (60.5% versus 31.8%, *P* = 0.002) rather than open-label. They also enrolled more patients per trial than did innovative drug trials (median [IQR], 358 [160 to 616] versus 219 [93 to 420], *P* = 0.02).

Compared to modified approvals, phase 1 or phase 1/2 pivotal trials were more frequently employed in innovative approvals (15.1% versus 0). Conversely, modified indications mostly received approval after phase 3 trials (76.7% versus 55.3%). Accordingly, the number of patients recruited for clinical trials of innovative drugs primarily in phase 1 or phase 2 was significantly fewer than that for clinical trials of modified new drugs primarily in phase 3. However, the number of patients allocated to the intervention group was almost the same (median [IQR], 152 [85 to 327] versus 179 [114 to 339]; *P* = 0.42), as was the median trial duration (median [IQR], 31.7 [22.4 to 48.7] versus 32.6 [23.8 to 50.6] months; *P* = 0.48).

Across the 128 pivotal clinical trials, the choice of comparator varied. Active comparator or placebo control was more common in modified trials than in innovative trials (active, 32.6% versus 22.4%; placebo, 53.5% versus 30.6%), while the use of no-treatment comparator was more common in innovative approvals than in modified ones (47.1% versus 14.0%). The choice of primary endpoints also significantly differed between the pivotal trials supporting innovative and modified approvals (ORR, 44.7% versus 11.6%; PFS, 22.4% versus 53.5%; OS, 14.1% versus 11.6%; *P* < 0.001) (Table 2).

**Table 2.** Characteristics of pivotal clinical trials supporting innovative and modified new anticancer drug approvals by the NMPA between 2016 and 2022

Characteristic	No. (%)			*P* value
	Trials, no. (128)	Innovative drugs (*n* = 85)	Modified new drugs (*n* = 43)	
No. of pivotal efficacy trials				0.001
1 pivotal trial	82 (64.1)	44 (51.8)	38 (88.4)	
2 pivotal trials	38 (29.7)	33 (38.8)	5 (11.6)	
3 pivotal trials	8 (6.3)	8 (9.4)	0 (-)	
Trial designs				<0.001
RCT	83 (64.8)	45 (52.9)	38 (88.4)	
Single-arm	45 (35.2)	40 (47.1)	5 (11.6)	
Phase				0.06
Phase 1	4 (3.1)	4 (4.7)	0 (-)	
Phase 1/2	9 (7.0)	9 (10.6)	0 (-)	
Phase 2	31 (24.2)	22 (25.9)	9 (20.9)	
Phase 2/3	4 (3.1)	3 (3.5)	1 (2.3)	
Phase 3	80 (62.5)	47 (55.3)	33 (76.7)	
Randomization				<0.001
Yes	83 (64.8)	46 (54.1)	37 (86.0)	
No	45 (35.2)	39 (45.9)	6 (14.0)	
Type of blinding				0.002
Double blind	53 (41.4)	27 (31.8)	26 (60.5)	
Open	75 (58.6)	58 (68.2)	17 (39.5)	
Comparator				0.001
Active	33 (25.8)	19 (22.4)	14 (32.6)	
No-treatment^a^	46 (35.9)	40 (47.1)	6 (14.0)	
Placebo	49 (38.3)	26 (30.6)	23 (53.5)	
Patients enrolled per trial, median (IQR)			
Overall	251 (107–430)	219 (93–420)	358 (160– 616)	0.02
Intervention group	178 (102–328)	152 (85–327)	179 (114– 339)	0.42
Clinical trial duration (months), median (IQR)		31.7 (22.4–48.7)	32.6 (23.8–50.6)	0.48
Primary endpoints				<0.001
ORR	43 (33.6)	38 (44.7)	5 (11.6)	
PFS	42 (32.8)	19 (22.4)	23 (53.5)	
OS	17 (13.3)	12 (14.1)	5 (11.6)	
Others	26 (20.3)	16 (18.8)	10 (23.3)	
RCT				0.38
ORR	5 (3.9)	3 (6.7)	2 (5.3)	
OS	16 (12.5)	11 (24.4)	5 (13.2)	
PFS	42 (32.8)	19 (42.2)	23 (60.5)	
Others	20 (15.6)	12 (26.7)	8 (21.1)	
Single-arm				0.17
ORR	38 (29.7)	35 (87.5)	3 (60.0)	
OS	1 (0.8)	1 (2.5)	0 (-)	
Others	6 (4.7)	4 (10.0)	2 (40.0)	

^a^ No treatment: Includes supportive therapy or standard care.

### Subgroup analysis of pivotal trial characteristics

Whether these differences in clinical trial designs were driven by the origin of anticancer drugs, the cancer type, or the regulatory characteristics (e.g., the frequency with which approvals use expedited regulatory programs) remains a question. We summarized and compared the characteristics of pivotal trials supporting innovative and modified new cancer indication approvals, mainly stratified by origin, cancer type, special regulatory program, and review time (Table 3). Significant differences were identified in the trial design and type of blinding between innovative and modified new anticancer drugs, whether imported or domestically produced. Additionally, significant differences were observed in study comparators among the imported anticancer drugs, with over half of the modified indications (54.2%) being supported by trials using a placebo comparator, in contrast to 33.3% of innovative indications. Fewer imported modified approvals than innovative approvals were supported by single-arm trials (4.2% versus 44.4%), and this trend was consistent in the use of no-treatment comparator (8.3% versus 44.4%). Differences were also observed in the pivotal trials supporting the approval of solid anticancer drugs in terms of trial design (*P* < 0.001), type of blinding (*P* = 0.001), and the use of comparators (*P* < 0.001) between innovative and modified approvals.

**Table 3.** Characteristics of pivotal trials supporting innovative and modified new anticancer drugs approved by the NMPA, overall and stratified by origin, cancer type, special regulatory program, and review time

Characteristic	Trials, no. (*n* = 128)	No. (%)						
		Trial design		Type of blinding		Comparator		
		RCT (*n* = 83)	Single-arm (*n* = 45)	Double-blind (*n* = 55)	Open (*n* = 75)	Active (*n* = 33)	No-treatment (*n* = 46)	Placebo (*n* = 49)
Origin								
Domestic								
Innovative	22	10 (45.5)	12 (54.5)	5 (22.7)	17 (77.3)	5 (22.7)	12 (54.5)	5 (22.7)
Modified	19	15 (78.9)	4 (21.1)	12 (63.2)	7 (36.8)	5 (26.3)	4 (21.1)	10 (7.0)
*P* value		0.03		0.009		0.07		
Imported								
Innovative	63	35 (55.6)	28 (44.4)	22 (34.9)	41 (65.1)	14 (22.2)	28 (44.4)	21 (33.3)
Modified	24	23 (95.8)	1 (4.2)	14 (58.3)	10 (41.7)	9 (37.5)	2 (8.3)	13 (54.2)
*P* value		<0.001		0.05		0.007		
Cancer type								
Solid tumor								
Innovative	58	33 (56.9)	25 (43.1)	20 (34.5)	38 (65.5)	15 (25.9)	25 (43.1)	18 (31.0)
Modified	33	33 (100.0)	0 (-)	23 (69.7)	10 (30.3)	12 (36.4)	1 (3.0)	20 (60.6)
*P* value		<0.001		0.001		<0.001		
Hematologic malignancies								
Innovative	27	12 (44.4)	15 (55.6)	7 (25.9)	20 (74.1)	4 (14.8)	15 (55.6)	8 (29.6)
Modified	10	5 (50.0)	5 (50.0)	3 (30.0)	7 (70.0)	2 (20.0)	5 (50.0)	3 (30.0)
*P* value		0.76		>0.99		>0.99		
Special regulatory program								
Priority review								
Innovative	71	33 (46.5)	38 (53.5)	22 (31.0)	49 (69.0)	12 (16.9)	38 (53.5)	21 (29.6)
Modified	26	21 (80.8)	5 (19.2)	14 (9.6)	12 (16.4)	10 (38.5)	5 (19.2)	11 (42.3)
*P* value		0.003		0.04		0.007		
Special review								
Innovative	14	10 (71.4)	4 (28.6)	7 (50.0)	7 (50.0)	3 (21.4)	4 (28.6)	7 (50.0)
Modified	6	4 (66.7)	2 (33.3)	3 (50.0)	3 (50.0)	1 (16.7)	2 (33.3)	3 (50.0)
*P* value		>0.99		>0.99		>0.99		
Conditional approval								
Innovative	41	10 (24.4)	31 (75.6)	7 (17.1)	34 (82.9)	3 (7.3)	31 (75.6)	7 (17.1)
Modified	11	7 (63.6)	4 (36.4)	5 (45.5)	6 (54.5)	2 (18.2)	4 (36.4)	5 (45.5)
*P* value		0.04		0.11		0.06		
Review time (months), median (IQR)								
Innovative	85	13.07 (10.0–16.3)	11.03 (9.07–14.0)	12.63 (8.93–16.13)	11.93 (9.60–15.30)	13.17 (11.93–16.47)	11.03 (9.07–14.00)	12.63 (8.93–16.93)
Modified	43	9.5 (8.3–11.4)	16.7 (9.3–22.1)	9.42 (8.02–11.38)	9.87 (8.53–18.38)	9.87 (6.93–13.61)	13.28 (8.99–20.89)	9.5 (8.57–11.40)
*P* value		<0.001	0.43	0.006	0.55	0.06	0.67	0.008

For solid tumors, all modified approvals (100%) were based on RCTs, while about half of the innovative approvals (43.1%) were based on single-arm trials. However, for hematologic malignancies, no difference was observed in these trial designs, as almost all modified and innovative approvals used similar designs in terms of trial design, type of blinding, and comparator. A comparable percentage of innovative and modified indications granted special review were supported by similar trial designs. Among the indication approvals granted priority review, statistically significant changes were observed in the proportion supported by different trial designs. For the indication approvals granted conditional approval, the use of single-arm design in innovative trials was significantly higher than in modified ones (75.6% versus 36.4%, *P* = 0.04). It was also observed that modified approvals based on RCT, double-blind, placebo design were reviewed in less time than the corresponding innovative approvals (*P* < 0.01).

The scale of enrolled patients and duration of pivotal trials among domestic innovative and modified approvals did not reveal significant discrepancies (Table 4). However, the scale of enrolled patients for imported modified approvals was significantly larger that for imported innovative approvals (median [IQR], 395 [316 to 786] versus 230 [93 to 431]; *P* = 0.002). Likewise, trials on drugs for solid tumors among modified approvals were larger than those in the innovative ones (median [IQR], 392 [243 to 703] versus 255 [112 to 547]; *P* = 0.04). There were no significant differences in the number of patients or trial duration between the innovative or modified trials, regardless of whether they were designated as special review, priority review, or conditional approval.

**Table 4.** Number of patients enrolled and duration of pivotal trials supporting the innovative and modified approvals by the NMPA, overall and stratified by origin, cancer type, special regulatory program, and review time

Characteristic	Treated patients, median (IQR), no.		Duration, median (IQR), months
	Overall	Intervention	
Origin			
Domestic			
Innovative	175 (92–408)	145 (90–276)	29.77 (18.33–51.42)
Modified	233 (108–358)	151 (84–196)	34.80 (23.80–49.67)
*P* value	0.79	0.76	0.36
Imported			
Innovative	230 (93–431)	153 (82–381)	31.93 (22.67–48.80)
Modified	395 (316–786)	261 (148–477)	32.52 (22.94–51.38)
*P* value	0.002	0.19	0.88
Cancer type			
Solid tumor			
Innovative	255 (112–547)	234 (119–421)	32.70 (22.66–47.33)
Modified	392 (243–703)	214 (136–424)	36.60 (27.85–51.12)
*P* value	0.04	0.94	0.18
Innovative	102 (83–280)	93 (41–165)	29.85 (19.39-58.40)
Modified	121 (84–316)	106 (60–178)	23.37 (16.81-41.54)
*P* value	0.67	0.62	0.47
Priority review			
Innovative	174 (87–405)	152 (85–302)	31.67 (22.64-47.33)
Modified	287 (117–490)	178 (91–338)	31.52 (22.38-49.90)
*P* value	0.13	0.69	0.87
Special review			
Innovative	411 (160–628)	249 (119–344)	24.62 (17.22-35.03)
Modified	106 (80–413)	91 (57–318)	19.22 (15.06-19.22)
*P* value	0.08	0.16	0.55
Innovative	113 (73–197)	85 (40–200)	33.47 (17.30-58.77)
Modified	120 (86–355)	116 (55–228)	32.07 (20.50-39.57)
*P* value	0.41	0.62	0.65

### Treatment outcomes in clinical trials

**Fig. 2.**
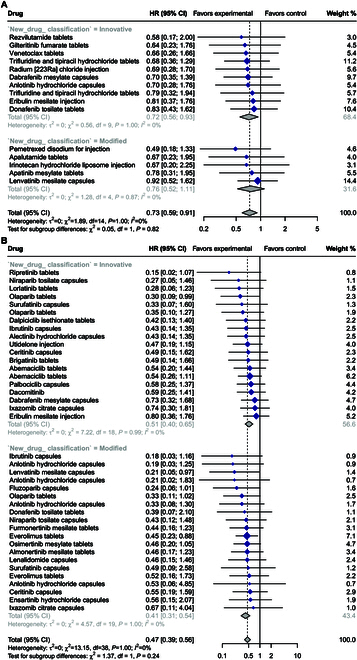
Forest plot of all RCTs with data on OS (A) and PFS (B) used for approval of innovative and modified new anticancer drugs between 2016 and 2022.

Out of the 128 pivotal trials, 83 (65%) were RCTs, among which 15 RCTs reported treatment outcomes for OS, and 39 RCTs reported PFS in either regulatory review documents or ClinicalTrials.gov. The pooled HR of OS in the meta-regression analysis was 0.73 [95% confidence interval (CI), 0.59 to 0.91; *I*^2^ = 0%; *P* > 0.99]. The innovative drugs had a higher clinical value in reduction in the risk of death with an HR for OS of 0.72 (95% CI, 0.56 to 0.93; *I*^2^ = 0%) compared with the modified new drugs’ HR for OS of 0.76 (95% CI, 0.52 to 1.11; *I*^2^ = 0%) (Fig. 2A). However, there was no statistically significant (*P* = 0.82) difference in the pooled HRs for OS between modified drugs and innovative drugs.

The pooled HR for PFS in the meta-regression analysis was 0.47 (95% CI, 0.39 to 0.56; *I*^2^ = 0; *P* > 0.99). Treatment outcomes for PFS gain were higher in modified new drugs’ indications (HR, 0.41; 95% CI, 0.31 to 0.54; *I*^2^ = 0) than in innovative drugs’ indications (HR, 0.51; 95% CI, 0.40 to 0.65; *I*^2^ = 0) (Fig. 2B). Notably, the difference between trial types was not statistically significant (*P* = 0.24).

Overall, meta-analyses of both endpoint OS and PFS showed that the efficacy outcomes of modified new anticancer drugs were not statistically significant from innovative anticancer drugs.

## Discussion

To the best of our knowledge, this is the first study to analyze the characteristics of pivotal trials supporting innovative and modified new anticancer drugs in China. In this study, a total of 77 new anticancer drugs were assessed for 107 indications supported by 128 pivotal trials approved by the NMPA since the implementation of the new drug registration classification in 2016. The results revealed that the pivotal trials supporting the approval of innovative drugs were more likely to be single-arm trials. In contrast, modified new anticancer drugs tended to be more rigorous and larger in scale, which had larger sample sizes and were more often conducted as double-blind RCTs compared to the trials supporting the innovative drugs’ initial approvals in China.

In the United States, the original indication and supplemental indication of the new active ingredients were approved through the same NDA pathway as NDA and sNDA (supplemental new drug application) [29]. However, the registration classification in China is different from the United States. The original drug approvals of new active ingredients with the initial indication were approved via the innovative drugs (including Class 1 and Class 5.1 pathway), and the supplemental indication approvals were approved via the modified new drugs (including Class 2.4 and Class 5.1 pathway) in China. To date, there are numerous studies conducted concerning the evidence or clinical value of initial and supplemental indications of anticancer drugs approved in the United States [27–29,36–38]. For example, Michaeli et al. [19] reported that initial approvals were often supported by single-arm trials, and supplemental indications were more often supported by rigorous trial design for RCTs. Our findings are similar to Michaeli’s finding regarding the clinical benefits associated with the distinction between initial and supplemental indications in the United States.

Pivotal trials for modified new anticancer drugs exhibited more characteristics associated with rigorous analysis than those for innovative drugs. This difference may be attributed to the relative lack of clinical urgency in modified approval, and this trend is similar to the differences between pivotal trials for biosimilar and originator [39]. By contrast, innovative approvals authorized with priority review or conditional approval were more likely supported by single-arm trial designs with lower phase (phase 1 or phase 1/2), nonrandomized, open-label, no-treatment comparator, the choice of ORR as primary endpoint, and fewer enrolled patients. This suggests that the strength of the aggregated evidence for innovative approvals was weaker than that of the modified ones, possibly indicating that innovative approvals were based on insufficient clinical data [27]. In fact, this characteristic aligns with the development strategy of pharmaceutical companies to accelerate the approval of anticancer drugs with high unmet need, as well as the regulatory trend toward enhancing patient access to cancer therapies. Furthermore, this result also indicates that approval of innovative drugs with nonrobust clinical evidence may overestimate clinical efficacy outcomes [19], which may mislead the physicians and patients who believe the robustness of drugs approved by the regulatory authorities.

The special regulatory programs were introduced to accelerate drug development and review in China. The conditional approval program is granted to drugs intended to treat serious or life-threatening ailments based on surrogate endpoints or intermediate results [6]. This means that drugs receiving special regulatory programs may offer significant efficacy or safety improvements relative to the standard of care in areas with high unmet clinical need [11]. Michaeli et al. [27] observed that the initial approvals were more likely to be granted special regulatory program by the U.S. Food and Drug Administration (FDA) than supplementary indications. Similar to this research, our study also revealed that innovative approvals had a greater likelihood of being granted special regulatory programs than modified ones. The possible reason may be that innovative drugs are always being considered as initial approvals that could provide greater clinical benefits for patients with a high unmet need than modified indications. These changes likely contributed to more flexible standards for approval, in line with previous studies [3,29,40].

According to the new drug registration classification, innovative drugs are defined as New Molecular Entities (NMEs) with initial indication approvals. Drug repurposing (or repositioning) refers to finding new indications for existing drugs, which may also include new dosage, new formulation, and a new method of use or new patient population [41]. In China, repurposed drugs are classified as modified new drugs by regulatory authorities. Modified new drugs are required to have significant clinical advantages in several policy documents [5,7,42]. However, there are no universal strict rules specifying the precise requirements for these clinical advantages. Our results suggested that most of the modified new drug approvals, based on more robust, later-stage phase and larger-scale trials, were NMEs with added new indications but without any change in the formulation or dosage (Table S4). There are some differences in clinical value outcomes. Our study did not find a statistically significant difference in treatment outcomes for OS or PFS improvement between innovative and modified drug indications. In contrast, a study conducted by Michaeli et al. reported a progressive loss of clinical benefit across indication extensions. This could be attributed to the pivotal trials for innovative drugs that are frequently conducted in small, specific patient subgroups characterized by positive biomarkers and severe diseases [19,43]. Additionally, this may reflect the stricter regulatory requirements of significant clinical advantages for modified new drugs in China. Regulatory agencies use special review pathways to prioritize resources toward indications believed to offer significant value to patients [27]. Therefore, it will be necessary for regulatory authorities to consider whether it is appropriate to include newly added indications of NMEs in the classification of modified new drugs.

With rapidly increasing launch prices for anticancer drug, access to high-quality information about the benefits and risks of new therapies is important, which allows patients to make truly informed treatment decisions [44]. These results have implications for patients and clinicians when making decisions regarding whether to use the initial innovative products, which were based on smaller and less rigorous pivotal trials. As some studies have suggested, less robust preapproval testing raises questions about the efficacy and clinical value of newly approved drugs [45].

All of these findings suggest the role of special regulatory programs in accelerating approvals for innovative cancer indications based on flexible, less robust trials or those using surrogate endpoints. Previous research studies have underscored the need for the drug regulatory authorities such as FDA to implement safety measures after a drug or biologic product is marketed, including issuing a boxed warning or timely confirmatory studies, especially for those that have been granted expedited approval designation or underwent priority review process [28,46,47]. Likewise, the NMPA will require post-marketing confirmatory studies for drugs granted special regulatory programs, especially for conditional approvals. Ongoing improvements in post-marketing surveillance methods, along with stricter study conduct and reporting standards, may enhance the quality of clinical evidence regarding the safety and efficacy of medications after approval, informing patients, healthcare providers, and regulators [42]. However, the post-marketing confirmatory studies are submitted through supplemental application in China [6], of which the regulatory review documents are not yet publicly available. Consequently, it is difficult to assess the implementation of clinical trials after conditional approval for marketing. Therefore, it is recommended that the NMPA improves the transparency of post-marketing studies for conditional approvals.

### Limitation

This study has several limitations. First, it included only chemical anticancer drugs. Conclusions drawn from this study are limited to anticancer drugs and cannot be extended to the full range of therapeutic agents. Second, we excluded 2 modified new drugs that were supported only by bioequivalence trials. We believe that this exclusion will not impact our study results, as these cases only accounted for less than 5% (2.6%, 2/77). Third, we only included clinical trials identified as pivotal trials in our study. Although studies such as dose-range trials and clinical pharmacology studies may contribute to regulatory review, we did not include these nonpivotal studies as they were not considered decisive factors in approval decisions. Finally, further research is necessary to be conducted to analyze the differences between the full range of new drugs approved in China.

## Conclusion

In conclusion, the findings of this study suggest that the quality of clinical trial evidence supporting the approval of innovative and modified new drugs varied. Pivotal trials for modified new anticancer drugs were more likely to be RCTs with rigorous designs and larger sample sizes, while the pivotal trials supporting innovative approvals exhibited more flexible and less robust trial designs. However, despite these differences, the efficacy outcomes were statistically indistinguishable between innovative and modified new anticancer drugs.

## Ethical Approval

Our study used only published data and did not involve any confidential patient information. The requirement for ethical approval and informed consent was waived for this study.

## Data Availability

L.F., X.P., and Y.C. had full access to all the data in the study and take responsibility for the integrity of the data and the accuracy of the data analysis. Data supporting the findings were collected from publicly available database and are included in this article and the supplementary files.
