# Supplementary material for: Comparative Assessment of Pivotal Trials Supporting the Indication Approvals of Innovative and Modified New Anticancer Drugs in China, 2016–2022
Source: Health Data Sci. 2025 May 2;5:0263. doi: 10.34133/hds.0263 (PMC12046133; doi:10.34133/hds.0263)
Supplement: Supplementary 1 — Tables S1 to S4 [file hds.0263.f1.zip › Supplementary Materials-for HDS.docx]

**Supplementary Materials**

**Assessment of pivotal trials supporting the approval of innovative vs. modified new anticancer drugs in China** **between 2016 and 2022**

**Table S1.** Summary of landmark documents related to regulatory reform in China

**Table S2.** The concepts of innovative and modified new drugs

**Table S3.** Expedited regulatory programs in China

**Table S4**. The categorization of 107 indications (107 NDAs)

**Table S1.** Summary of landmark documents related to regulatory reform in China [1-4]

| **Initiation date** | **Issuing Organization** | **Policy Title** | **Measures to drug registration** |
| --- | --- | --- | --- |
| 2015-08 | The State Council | Opinions on the Reform of Review and Approval System for Drugs and Medical Devices ([2015] No.44) | Introduced the categorization of drugs into new and generic drugs, further classifying new drugs into innovative and modified new drugs. |
| 2016-03- | National Medical Products Administration | Work Plan for the Reform of Chemical Drug Registration Classification ([2016] No.51) | Redefined the classification of chemical drug registration |
| 2020-03- | The State Administration for Market Regulation | Provisions for Drug Registration | Redefined the categorization of drugs into innovative drugs, modified new drugs, and generic drugs, etc. |
| 2020-06 | National Medical Products Administration | Requirements for Registration Classification and Application Dossiers of Chemical Drugs | Further clarified and refined the registration classification of chemical drugs |

**Table S2**. The concepts of innovative and modified new drugs [2]

| **Categories of NDAs (NMPA)** |  | **Definition** |
| --- | --- | --- |
| **Class 1** (Innovative drugs) |  | **Innovative drugs** that have not been marketed in China or overseas. They refer to drugs that contain new compounds with clear structures and pharmacological effects, and have clinical values. |
| **Class 2** (Modified new drugs) |  | **Modified new drugs** that have not been marketed in China or overseas. They refer to drugs that have their structure, dosage form, formulation and process, route of administration and indications optimized on the basis of known active ingredients and have significant clinical advantages. |
|  | Class 2.1 | Drugs that contain an optical isomer of known active ingredients obtained by resolution or synthesis, or esterification of known active ingredients, or salification of known active ingredients (including salt containing hydrogen bonds or coordination bonds), or change in acid group, basic group, or metallic element of known active ingredients of salt, or formation of other non-covalent bond derivatives (e.g., complex, chelate or clathrate), and have significant clinical advantages. |
|  | Class 2.2 | Drugs that contain known active ingredients with new dosage form (including new drug delivery system), new formulation process or new route of administration, and have significant clinical advantages. |
|  | Class 2.3 | New compound preparations that contain known active ingredients and have significant clinical advantages. |
|  | Class 2.4 | Drugs for new indications that contain known active ingredients. |
| **Class 5** (Innovative drugs, Modified new drugs, Generic drugs) |  | Drugs that have been **marketed overseas** and are under application for being marketed in China. |
|  | **Class 5.1** | **Original drugs and modified drugs** that have been marketed overseas and are under application for being marketed in China. Modified drugs shall have obvious clinical advantages. |
|  | Class 5.2 | **Generic drugs** that have been marketed overseas and are under application for being marketed in China. |

**Table S3.** Expedited regulatory programs in China [1,5]

| **Items** | **Effective period** | **Application standards and requirements** |
| --- | --- | --- |
| Special Review | 2007-2020 | A program issued in the 2007 PDR and applicable for new drugs that not yet launched in any countries. This program was removed from the 2020 PDR. |
| Priority Review and Approval | 2015-present | A program introduced by the former CFDA in November 2015 and issued in the 2020 PDR. This program applies to primarily new drugs that showing substantial clinical benefits, for urgently needed conditions and diseases such as major infectious diseases and rare diseases, and new or modified drugs for pediatrics. |
| Conditional Approval | 2017- present | A program introduced by the CFDA in December 2017 and issued in the 2020 PDR. CA applies to drugs used for treating serious life-threatening diseases, urgently needed for public health, or vaccines urgently needed for response to critical public health emergencies. |
| Breakthrough Therapy | 2020- present | A designation issued in the 2020 PDR and applicable to new drugs used for the prevention and the treatment of diseases that seriously endanger life or affect the quality of life, for which there is no effective prevention and treatment, or, compared with existing measures of treatment, there is sufficient evidence proving the obvious clinical advantages. |
| Special review and approval | 2020- present | A designation issued in the 2020 PDR and applicable to therapeutic and prophylactic drugs that are developed when a public health emergency breaks out. |

PDR, Provisions for Drug Registration; CFDA, China Food and Drug Administration

**Table S4**. The categorization of 107 indications (107 NDAs)

| **Anticancer drug type** | **Categories of NDAs** | **Types of NDA applications** | **No. of NDAs** |
| --- | --- | --- | --- |
| Innovative (64) | Class 1 | Initial indication | 23 |
|  | Class 5.1 | Initial indication | 41 |
| Modified (43) | Class 2.2 | New dosage form | 3 |
|  | Class 2.4 | New indication | 21 |
|  | Class 1 | New indication | 3 |
|  | Class 5.1 |  |  |
|  |  | New indication | 14 |
|  |  | New dosage form | 1 |
|  |  | New formulation | 1 |

NDA, new drug application.

**Reference**

1 State Administration for Market Regulation. Provisions for Drug Registration; 2020. <http://english.nmpa.gov.cn/2022-06/30/c_785628.htm>. Accessed 2023/2/4, 2023.

2 National Medical Products Administration. NMPA Issues Requirements for Registration Classification and Application Dossiers of Chemical Drugs; 2020. <http://english.nmpa.gov.cn/2020-06/30/c_528659.htm>. Accessed 2023/2/4, 2023.

3 Xu L, Gao H, Kaitin KI, Shao L. Reforming China's drug regulatory system. *Nat Rev Drug Discov*. 2018;17(12):858-859.

4 Work Plan for the Reform of Chemical Drug Registration Classification; 2016. <https://www.nmpa.gov.cn/yaopin/ypggtg/ypqtgg/20160309151801706.html>. Accessed 2023/2/4, 2023.

5 Liu Y, Zhang N, Xie C, Jiang Y, Qin Y, Zhou L, Fan Y, Ren L, Yin C, Yang H, et al. Evolution of drug regulations and regulatory innovation for anticancer drugs in China. *Acta Pharmaceutica Sinica B*. 2022;12(12):4365-4377.
